# The effect of daily mean temperature on hand, foot and mouth disease and the source of regional heterogeneity in Chongqing, China, 2010–2019

**DOI:** 10.1265/ehpm.22-00133

**Published:** 2022-12-15

**Authors:** Xinyi Deng, Zhiyi Chen, Yang Zou, Ying He, Saijuan Chen, Qiuting Wang, Dianguo Xing, Yan Zhang

**Affiliations:** 1 School of Public Health, Research Center for Medicine and Social Development, Innovation Center for Social Risk Governance in Health, Research Center for Public Health Security, Chongqing Medical University, Chongqing, China; 2 Office of Health Emergency, Chongqing Municipal Health Commission, Chongqing, China

**Keywords:** Hand, foot and mouth disease, Temperature-disease relationship, Distributed lag non-linear model, Regional heterogeneity

## Abstract

**Background:**

Hand, foot and mouth disease (HFMD) is a serious infectious disease which has become a public health problem. A multi-regional study was conducted in this study to explore the relationship between temperature and HFMD in different regions and the source of heterogeneity, and further detect the effect modifiers such as socio-economic factors, medical and health factors and meteorological factors.

**Methods:**

The data on daily reported HFMD cases and meteorological data from 2010 to 2019 in Chongqing were collected. Thirty-eight districts and counties of Chongqing were divided into 6 regions. The distributed lag nonlinear model (DLNM) was applied to assess the effect of daily mean temperature on HFMD at region level with the pooled effect estimates from multivariate meta-regression model analysis. Stratified analyses by gender, age and children’s type were also conducted. Potential modifiers were considered in meta regression to explore the source of heterogeneity.

**Results:**

There were nonlinear relationships with an inverted V-shape between temperature and HFMD. A maximum cumulative relative risk (CRR) of 1.22 (95% confidence interval (CI): 1.12–1.34) peaked at 23.8 °C, and the risk appeared immediately and lasted for the whole 14 days. Compared with other groups, warm temperature had a stronger effect on children aged 0–1 and scattered children, while cold temperature had a stronger effect on female, children aged 3–6 and childcare children with an M-shape. We found that socio-economic factors, medical health factors and meteorological factors were significantly associated with heterogeneity. Density of medical technical personnel, urbanization rate and density of health care institutions were the main modifiers for explaining heterogeneity of 26.10%, 24.90% and 24.86% respectively which were revealed by meta-analysis.

**Conclusions:**

There was a significant nonlinear correlation between temperature and HFMD. Compared with other groups, children aged 0–1 and scattered children were more susceptible to warm temperature, while female, children aged 3–6 and childcare children were more susceptible to cold temperature. Socio-economic factors, medical health factors and meteorological factors may be the source of the heterogeneity. Therefore, local governments should consider different temperature–HFMD relationships between different regions and populations when formulating appropriate preventive measures.

**Supplementary information:**

The online version contains supplementary material available at https://doi.org/10.1265/ehpm.22-00133.

## Graphical Abstract

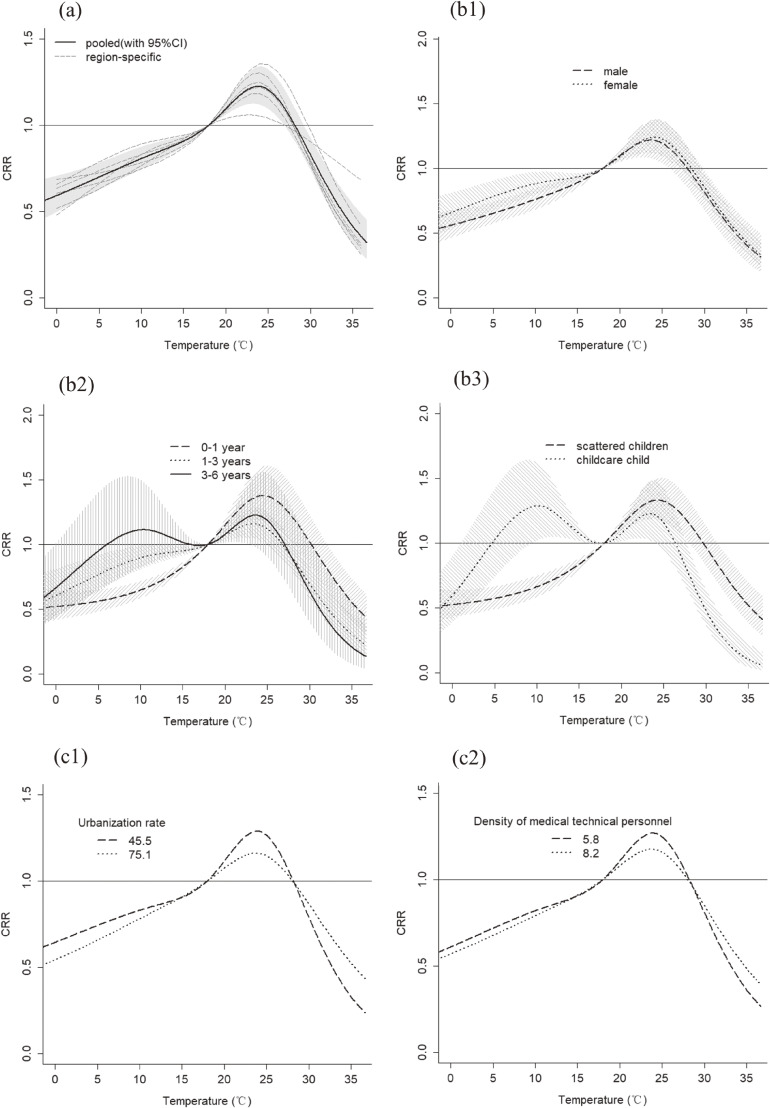

## 1. Introduction

Nowadays, climate change is considered as one of the greatest challenges threatening human health [1]. Controlling the negative effects of climate change has become a common goal in the world. In recent years, the incidence rate of some infectious diseases closely related to meteorological factors in China is still at a high level [2]. Due to the increasing social and economic burden caused by meteorological factors [3], more and more attention has been paid to the adverse effects of meteorological factors on human health [4].

Hand, foot and mouth disease (HFMD) is one of the legal class C infectious diseases in China which is caused by a variety of enterovirus infections [5]. The disease is highly infectious and can be transmitted through direct contact, droplets, fecal mouth and other means [6]. In recent years, its incidence rate has been in the first place of class C infectious diseases all the year round [5], which has become one of the public health issues of great concern in China. Previous studies have shown that the average temperature is the most important meteorological factor which can influence the occurrence and prevalence of HFMD by affecting multiple aspects such as pathogens, hosts, and human behaviors [7]. A study has applied a generalized additive model to estimate the effects of meteorological factors on HFMD, and found that there was a correlation between temperature and HFMD [8]. A study in Beijing used a case-crossover design combined with distributed lag nonlinear model (DLNM), and further found that there was a nonlinear relationship between temperature and HFMD [9], but the results of studies in different regions were different and controversial [10]. For example, some studies in Japan [11], Taiwan [12], and mainland China [13] showed an increased risk of HFMD at moderately high temperatures but a decreased risk at extremely hot temperatures, approximating an inverted V-shape, while a study in Singapore [14] showed a threshold and J-shaped relationship between temperature and HFMD. Besides, some studies in eastern China found that the exposure-response relationship between temperature and HFMD was non-linear with an approximate M-shape [15, 16]. The above studies were mainly focused on a single city or region, and the differences in the results might be attributed to the climatic conditions, socio-economic factors and demographic characteristics of different regions [17]. At present, several studies have begun to explore the heterogeneity of findings. A multi-site environmental epidemiology study [18] showed that spatial heterogeneity in the temperature-HFMD relationship could be well explained by location-specific humidity; Xiao [18] found that 68.5% of the variations of city-specific estimates was attributable to heterogeneity and identified rainfall and altitude as the two main effect modifiers; Guo [19] showed geographic variations among the cities in Guangdong which was significantly associated with city’s latitude and longitude with an explained heterogeneity of 32%; Xu [20] also found that latitude was the main factor that reduced the heterogeneity to 69.28%. However, these studies mostly explored the source of heterogeneity from the perspectives of climatic and geographical factors, but lacked in-depth exploration of heterogeneity from the perspectives of socio-economic factors and medical and health factors.

Chongqing is located in the southwest of China, in the middle and upper reaches of the Yangtze River. It is dominated by mountainous terrain. Affected by the terrain drop, the climate and economic development of each region in Chongqing are significantly different, with the typical characteristics of a dichotomy between a large city and a large rural area. Based on the above, the internal incidence of infectious diseases in the Chongqing often shows strong heterogeneity on the basis of commonality. Therefore, in order to analyze the influence of temperature on HFMD and explore the source of its heterogeneity, this study took Chongqing as the research area. According to the economic and climatic characteristics, Chongqing were divided into 6 regions, and a multi-region analysis was conducted. This study aimed to assess the impact of temperature on HFMD and to examine potential effect modification by socio-economic factors, medical and health factors and meteorological factors in six regions in Chongqing, China, with DLNM and multivariate meta-regression model analysis. The findings can provide useful information to the development and implementation of appropriate regional intervention strategies.

## 2. Materials and methods

### 2.1 Study area

**Fig. 1:**
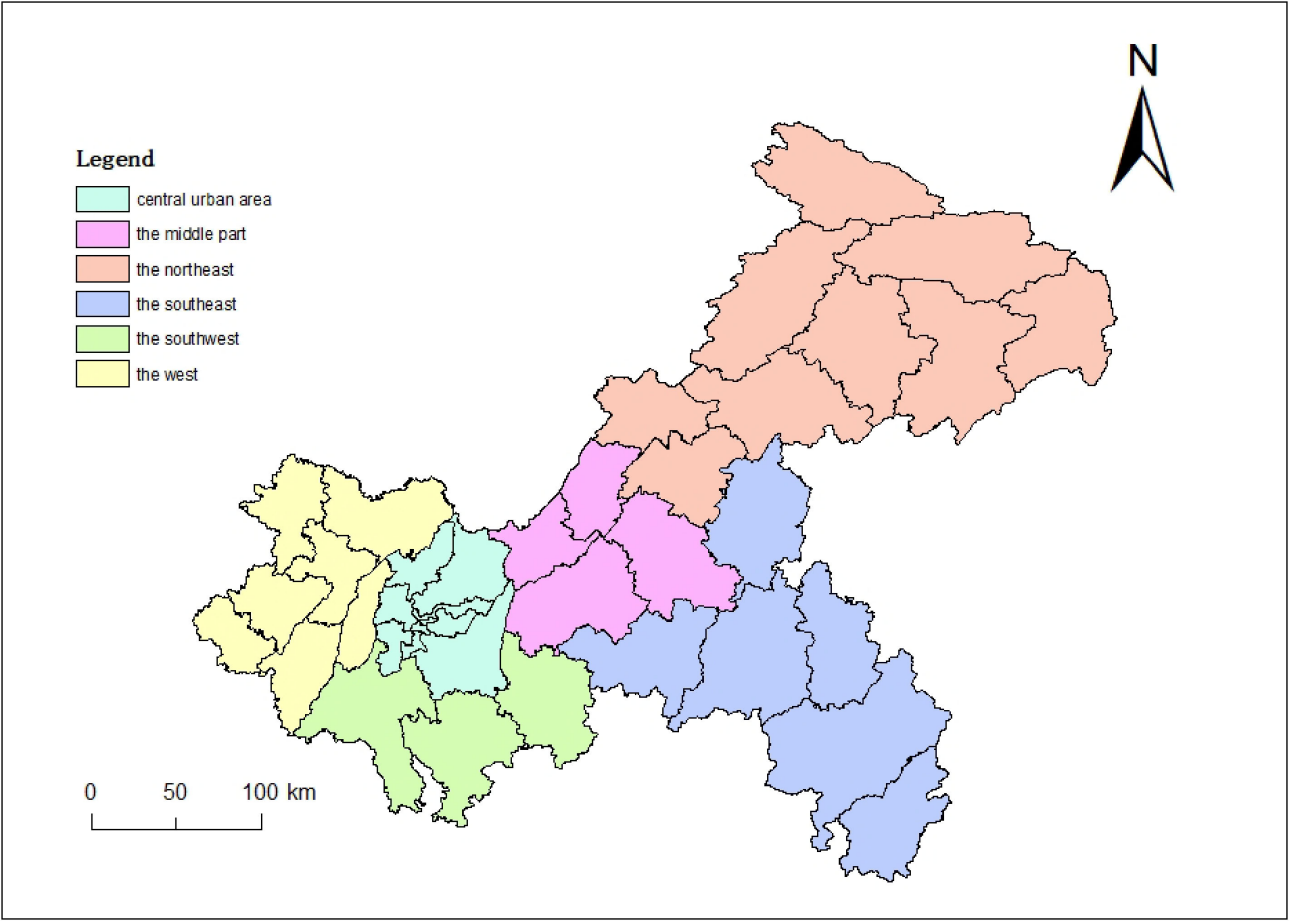
Distribution of six regions in Chongqing.

Chongqing is located in the southwest of China, with a total area of 82,400 km^2^, of which the mountainous area accounts for about 75% of the total area. Chongqing has 38 districts/counties and a total population of 31.2 million by the end of 2019. According to the geographical features, meteorological characteristics [21], economic development level and morbidity characteristics [22] of HFMD, we divided the 38 districts and counties of Chongqing into 6 regions as the focus of the study (Fig. 1), including central urban area (including 9 districts such as Yuzhong District), the west (including 7 districts/counties such as Dazu District), the southwest (including 3 districts/counties such as Qijiang District), the middle part (including 4 districts/counties such as Fuling District), the northeast (including 9 districts/counties such as Wanzhou District) and the southeast (including 6 districts/counties such as Qianjiang District). The central urban area and its surrounding areas have relatively flat terrain, developed economy and high urbanization rate. The east is mainly mountainous terrain, with poor economic level and low urbanization rate. The west has high relative humidity.

### 2.2 Data collection

The daily data of HFMD cases from 1 January 2010 to 31 December 2019 in 38 districts and counties of Chongqing were obtained from China Information System for Disease Control and Prevention (CISDCP, http://www.cdpc.chinacdc.cn), including the basic social demographic information of HFMD cases. In mainland China, HFMD has been monitored and reported as a class C notifiable disease since 2008. The diagnostic criteria of HFMD were based on the clinical criteria set by the Hand, Foot and Mouth Disease Control and Prevention Guide published by the National Health Commission of the People’s Republic of China [23]. A clinical HFMD case is defined as a patient with papular or vesicular rashes on hands, feet, mouth or buttocks, with or without fever. All clinical cases were reported to the web-based infection diseases monitor information system within 24 hours of diagnosis by use of a standardized form [24]. Daily meteorological variables, including mean temperature, precipitation, average relative humidity, sunshine hours and mean air pressure for the same period were collected from China Meteorological Data Sharing Service System. The resident population, urbanization rate, per capita GDP, population density, the number of health care institutions and medical technical personnel of 38 districts and counties were collected from Chongqing Statistical Yearbook [25], and these variables were obtained by calculating average values from 2010 to 2019.

### 2.3 Statistical analysis

We adopted a two-stage analytical method in this study. Firstly, we analyzed the region-specific estimates of temperature-HFMD relationship, and then we pooled the multi-region estimates and explored the heterogeneity.

In the first stage, a time-series DLNM with quasi-Poisson regression was applied to assess the effect of daily mean temperature on daily reported HFMD in the 6 regions respectively. The bi-dimensional exposure-lag-response relationship between temperature and HFMD was described through a cross-basis function [26], using natural cubic splines with 4 degrees of freedom (df) for the exposure-response relationship and natural cubic splines with 5 df for the lag-response relationship [27]. The optimal choices of df were determined by comparing the goodness of the model fits (measured by quasi akaike information, QAIC). Given the incubation and duration of HFMD, the maximum lag was set to 14 days to explore the whole lag structure of temperature effect. At the same time, spearman correlation and collinearity diagnosis were analyzed to explore and control the meteorological confounding factors. Spearman correlation was used to calculate the correlation between meteorological factors, and variance inflation factor (VIF) was used for collinearity diagnosis. Finally, the average relative humidity and mean air pressure were included in the model as hybrid variables which were modeled as natural cubic splines with 3 df. The natural cubic splines of calendar time with 8 df per year was used in the model to control the seasonality and long-term trends. Dow and Holiday are the categorical variables indicating the day of the week and the public holiday, respectively. The average value of temperature (18 °C) was defined as the reference for calculating relative risks. The model was defined by the following formula:

$$\begin{array}{rl} \log[\text{E}({\text{Y}}_{\text{t}})] & =\alpha +\sum_{\text{l}={\text{l}}_{0}}^{\text{L}}\gamma_{\text{l}}{\text{temp}}_{\text{t,l}}+\text{ns}(\text{date})+\text{ns}({\text{ruh}}_{\text{t}})\\ & \;+\text{ns}({\text{prs}}_{\text{t}})+\text{Dow}+\text{Holiday} \end{array}$$

Where E(Y_t_) represents the expected number of HFMD cases on day t. *α* is the intercept. *γ*_l_ represents coefficient; temp_t,l_ represents the cross-basic of temperature and time.

In the second stage, the region-specific estimates obtained from the first stage model were then combined through multivariate meta-analysis to reveal the pooled exposure–response relationship [28], and we used the maximum likelihood estimation to obtain estimates. Stratified analyses by gender, age and children’s type were also conducted. In order to explore heterogeneity, we first fitted a multivariate meta-regression model with intercept only allowing for heterogeneity modelled through random effects (intercept-only model). Then, we ran a single meta-predictor analysis by incorporating region-specific characteristics including socio-economic, medical and health, geographical and meteorological factors into the model separately (single meta-predictor models), and compared it to the intercept-only model, in which we allowed the heterogeneity to be partly explained by meta-variables and modelled as fixed effects. Because different meta-predictors can be correlated with each other, we only displayed meta-predictors which showed the largest impact in their own category to avoid the issue of collinearity and to identify the optimum subset of meta-predictors. These effects were tested through a multivariate Wald test. Residual heterogeneity was tested and reported by the multivariate extension of Cochran Q test and I^2^ statistic [29]. Goodness of fit test for the model was based on Akaike information criterion (AIC) and Bayesian information criterion (BIC).

All data analyses were performed in R 4.2.0, with the “dlnm” package to fit the DLNM and “mvmeta” package to conduct multivariate meta-analysis. Two-sided P values less than 0.05 were considered as statistically significant.

## 3. Results

### 3.1 Descriptive analysis

Table 1 summarized the basic characteristics of HFMD in 6 regions of Chongqing from 2010 to 2019, with a total of 461,782 cases. The average annual incidence in Chongqing was 152 per 100,000 persons in the whole population, with the annual incidence varying from 69 per 100,000 persons to 297 per 100,000 persons [see Additional file 1 Table S1]. A greater number of the patients were male, accounting for 58.2% of total cases. The male to female sex ratio was 1.39. The majority cases were under 1 year old and between 1 and 3 years old, accounting for 42.2% and 40.1%, respectively. HFMD cases were mainly scattered children, accounting for 65.2% of total cases.

**Table 1:** Distribution of HFMD cases in six regions of Chongqing from 2010 to 2019

**Region**	**Total**	**Male**	**Female**	**0–1 year**	**1–3 years**	**3–6 years**	**Scattered children**	**Childcare children**
Central urban area	228606	131739	96867	91671	94769	35024	137540	82867
The west	66334	38828	27506	30774	25673	8189	46035	18386
The southwest	27728	16276	11452	12890	10575	3570	19597	7341
The middle	25634	14950	10684	10683	10525	3760	16622	8286
The southeast	22407	13534	8873	10804	8184	2799	16834	4845
The northeast	91073	53273	37800	38081	35673	14045	64340	22973

Total	461782	268600	193182	194903	185399	67387	300968	144698

**Fig. 2:**
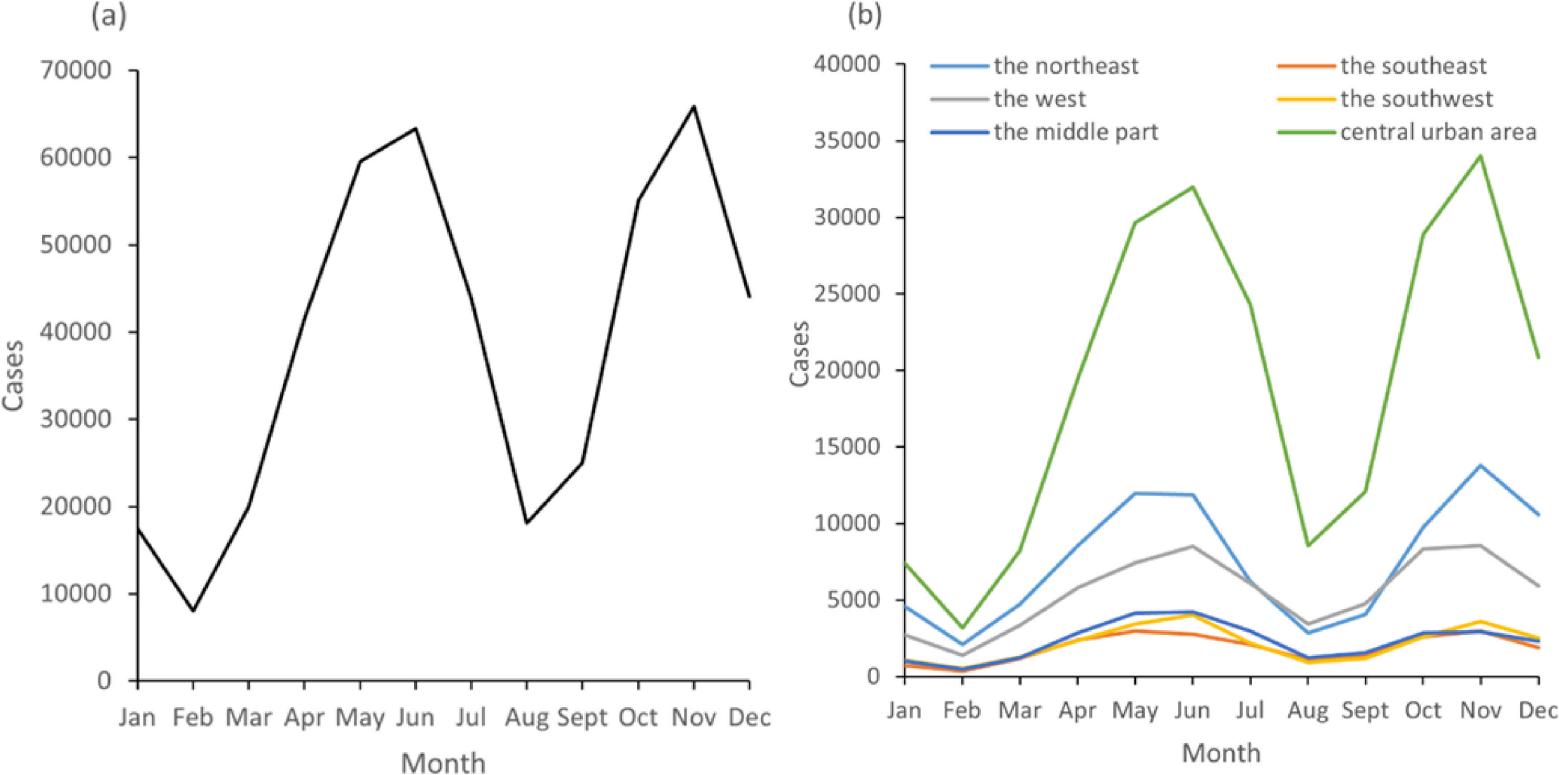
Distribution of HFMD cases in Chongqing from 2010 to 2019. (a) Monthly distribution of HFMD cases in Chongqing from 2010 to 2019. (b) Monthly distribution of HFMD cases in six regions of Chongqing from 2010 to 2019

Figure 2 showed the monthly and region-specified distribution of HFMD cases in Chongqing, respectively. It can be found that the first high-incidence periods of HFMD in Chongqing were mainly concentrated in late spring and early summer (April–July), and the second high-incidence periods were in autumn (October–December). The two peaks were June and November, with 63,327 cases (13.7%) and 65,843 cases (14.3%). The peaks were reached one month earlier (May) in the northeast and the southeast. Figure S1 [see Additional file 1] showed the distribution of HFMD cases by Year. Figure S2 [see Additional file 1] showed the distribution of HFMD cases by temperature in Chongqing from 2010–2019, and it can be found that the HFMD cases in Chongqing were mainly concentrated at 15–28 °C, with a peak at 23 °C.

Table 2 summarized the geographical and meteorological characteristics of the 6 regions in Chongqing. The socio-economic characteristics and medical and health characteristics of each region in this study were shown in Table 3.

**Table 2:** Geographical and meteorological information in six regions of Chongqing

**Region**	**Latitude** **(°N)**	**Longitude** **(°E)**	**Altitude** **(m)**	**Temperature** **(°C)**	**Precipitation** **(mm)**	**Humidity** **(%)**	**Sunshine hours (h)**	**Air pressure** **(hPa)**
Central urban area	29.35	106.28	259.1	19.08	3.29	74.75	2.81	983.22
The west	29.50	105.80	312.7	17.81	3.00	81.84	3.12	972.30
The southwest	29.09	106.27	258.1	18.43	2.93	77.92	2.89	971.83
The middle	29.51	107.24	334.1	18.61	3.00	76.88	3.21	974.57
The southeast	28.91	108.47	635.7	15.09	3.50	78.87	2.85	929.72
The northeast	30.74	108.78	243.3	18.90	2.94	72.26	3.45	985.83

**Table 3:** Socio-economic characteristics and medical and health characteristics in six regions of Chongqing

**Region**	**RP (× 10000)**	**UR (%)**	**Per capita GDP (RMB)**	**Population density (people per km^2^)**	**DHCI (per 10000 persons)**	**DMTP (per 1000 persons)**
Central urban area	825.20	91.22	106107	4611.71	2.669	11.52
The west	593.40	54.30	79762	609.37	0.406	6.10
The southwest	296.36	65.40	64114	319.56	0.223	5.86
The middle	318.16	58.37	73266	408.28	0.244	6.20
The southeast	284.59	43.27	47241	169.31	0.130	5.72
The northeast	689.91	46.79	45042	274.22	0.217	5.81

Note: RP, resident population; UR, urbanization rate; DHCI, density of health care institutions; DMTP, density of medical technical personnel.

### 3.2 Effect of daily mean temperature on HFMD

**Fig. 3:**
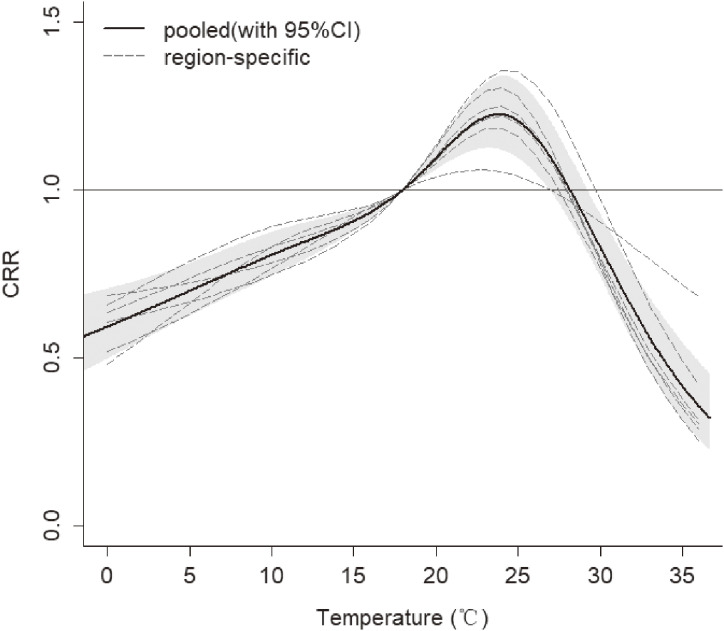
Pooled overall temperature–HFMD response curve based on 6 regions in Chongqing. The overall pooled estimate of cumulative relative risk is shown as smooth black lines and the pointwise 95% confidence intervals are shown in the gray regions. (on lag 0–14 days and reference temperature of 18 °C)

**Fig. 4:**
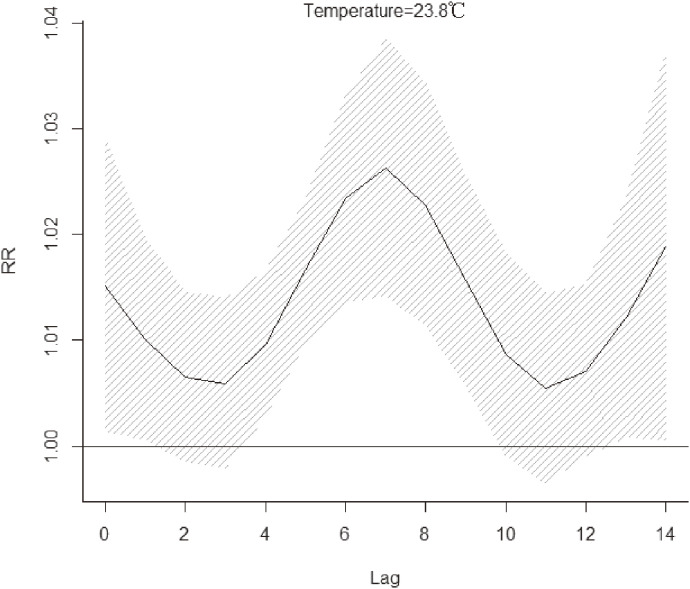
Relative risk of daily HFMD at 23.8 °C. (on lag 0–14 days and reference temperature of 18 °C)

Figure 3 revealed the cumulative relative risk (CRR) of daily HFMD associated with daily mean temperature on lag 14 days. The pooled overall cumulative effect revealed that the temperature-HFMD relationship was nonlinear with an approximately inverted V-shape. We choose average daily mean temperature (18 °C) as the reference temperature. The pooled overall cumulative effect started to rise until reached the highest CRR, which was 1.22 (95% confidence interval (CI): 1.12–1.34) over lag 0–14 days at 23.8 °C. Then the CRR decreased when temperature was higher than 23.8 °C. Between 18–28.1 °C, temperature was a hazard factor associated with HFMD. In other temperature ranges, temperature was a protective factor. Figure 4 illustrated the relative risk (RR) of HFMD at the temperature where the CRR peaked (23.8 °C). The associations of this temperature with HFMD appeared immediately and lasted for the whole 14 days, and peaked on the seventh day.

**Fig. 5:**
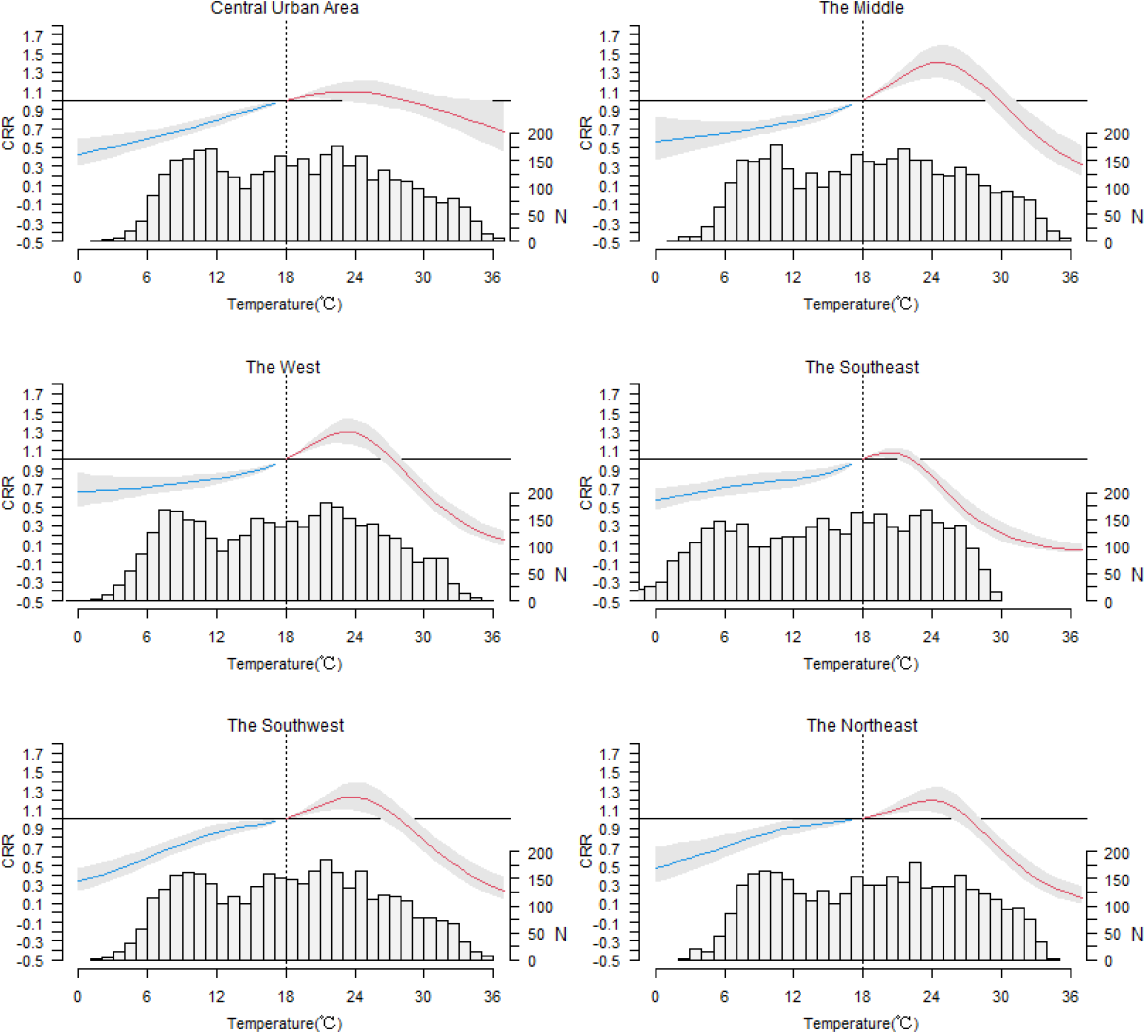
Cumulative relative risk of HFMD by daily mean temperature in 6 regions. (on lag 0–14 days and reference temperature of 18 °C)

Figure 5 showed the exposure-response relationships in each region. In the all regions, the effect of temperature showed an approximately inverted V-shape. However, the highest CRR varied from region to region which showed the great heterogeneity. The peaks of CRR of HFMD in the west and middle were highest, which were 1.30 (95% CI: 1.17–1.43) and 1.41 (95% CI: 1.24–1.59) respectively. While the peaks in the central urban area and the southeast were lowest, which were 1.09 (95% CI: 1.00–1.20) and 1.07 (95% CI: 1.02–1.12) respectively.

**Fig. 6:**
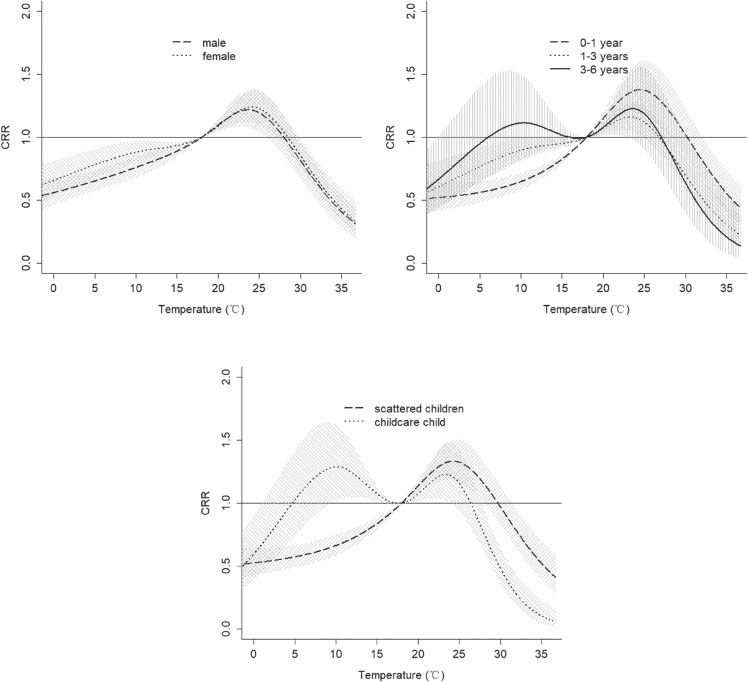
The CRR of mean temperature for gender-, age-, children’s type-specific HFMD cases over 14 days. (on lag 0–14 days and reference temperature of 18 °C)

Figure 6 presented the CRR of exposure to mean temperature for different people groups with HFMD over 14 days. The warm temperature had a similar effect on female and male, while the cold temperature had a stronger effect on female than male. The curves of temperature effect on HFMD in different aged groups were different. The effect of temperature in aged 0–1 showed an approximately inverted V-shape peaked at 24.5 °C, with CRR value: 1.38 (95% CI: 1.18–1.60), which was higher than the other aged group. However, the effect of temperature in children aged 3–6 showed an approximately M-shape, and temperature was a hazard factor associated with HFMD when temperature ranged from 6 °C to 18 °C. The temperature had a similar effect on childcare children and children aged 3–6, with an approximately M-shape. When temperature ranged from 5 °C to 18 °C, temperature was a hazard factor associated with HFMD, with CRR value: 1.29 (95% CI: 1.02–1.62) at 10.1 °C.

The results of random-effect meta-analysis (intercept-only) and multivariate meta-regression with a meta-predictor in the second stage were shown in Table 4. The heterogeneity among different regions was statistically significant based on the Cochran Q test (Q = 374.75, P < 0.01). I^2^ suggested that 73.32% of heterogeneity was due to actual difference of 6 regions. According to the P values of Wald test and I^2^, nine modifiers were associated with temperature-HFMD relationship, including longitude, urbanization rate, per capita GDP, density of health care institutions, density of medical technical personnel, temperature, humidity and sunshine hours, which were significantly related to the heterogeneity for decreasing I^2^. In particular, density of medical technical personnel could explain the largest proportion of heterogeneity with ΔI^2^ equal to 26.10%. Urbanization rate and density of health care institutions could explain heterogeneity of 24.90% and 24.86%, respectively. Besides, we displayed the modification effects of urbanization rate, density of medical technical personnel, sunshine hours and longitude, which could reduce the heterogeneity to 27.00%.

**Table 4:** Multivariate meta-regression models for regions

**Meta-predictors**	**Cochran Q test**		**I^2^ (%)**	**Model fits**		**Wald test**	
	
	**Q**	**p**		**AIC**	**BIC**	**stat**	**p**
Intercept-only model							
Intercept-only	374.75	<0.01	73.32	164.15	763.34		
Single meta-predictor models							
Latitude	311.68	<0.01	74.33	77.99	774.87	36.93	0.01
Longitude	292.64	<0.01	72.66	77.88	774.75	40.91	<0.01
Altitude	321.55	<0.01	75.12	77.09	773.96	28.54	0.10
UR	155.10	<0.01	48.42	71.86	768.73	134.92	<0.01
Per capita GDP	203.90	<0.01	60.77	76.03	772.90	67.69	<0.01
DHCI	155.22	<0.01	48.46	74.24	771.11	102.10	<0.01
DMTP	151.57	<0.01	47.22	73.23	770.10	117.94	<0.01
Temperature	286.02	<0.01	72.03	75.68	772.56	37.02	0.01
Humidity	282.45	<0.01	71.68	76.36	773.23	51.41	<0.01
Sunshine hours	249.10	<0.01	67.88	77.00	773.87	45.96	<0.01
Air pressure	312.80	<0.01	74.42	77.14	774.01	29.77	0.07
Multiple meta-predictors model							
UR, DMTP, Sunshine hours and longitude	27.40	0.12	27.00	141.62	1005.75	334.48	<0.01

Note: UR, urbanization rate; DHCI, density of health care institutions; DMTP, density of medical technical personnel.

**Fig. 7:**
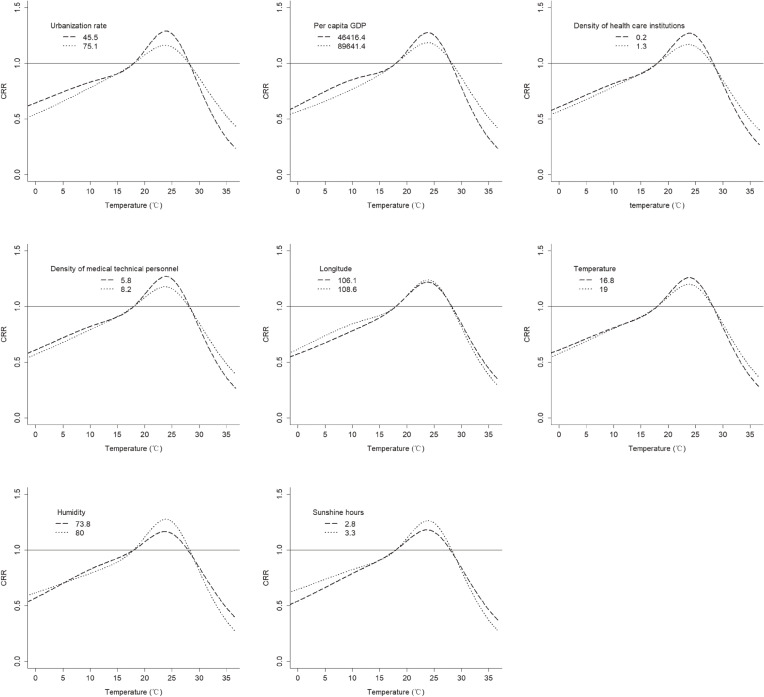
The cumulative effects at 12.5th and 75.5th of meta-predictors from meta-regression. The dotted black line and the dotted grey line represent the 12.5th and 75.5th of urbanization rate respectively.

Figure 7 showed the cumulative effects at 12.5th and 87.5th of meta-predictors from meta-regression. The results showed some differences in the effect of temperature on HFMD at different levels. In terms of economic factors, the warm temperature had a stronger effect on HFMD in areas with lower urbanization rate and lower per capita GDP; in terms of medical factors, the warm temperature had a stronger effect on HFMD in areas with lower density of health care institutions and lower density of medical technical personnel; in terms of geo-meteorological factors, the warm temperature had a stronger effect on HFMD with higher longitude, lower temperature, higher humidity, and longer sunshine hours.

## 4. Discussion

In this study, we found that the daily cases of HFMD in Chongqing had a semiannual peak with a relatively obvious seasonality. The first peak was concentrated in late spring and early summer (April–July), and the second peak was in autumn (October–December). This was because the climatic conditions during this period, such as temperature, humidity, and sunlight, were suitable for the survival and reproduction of pathogens. A study [30] showed that some potential factors, including the infectivity of pathogens in vitro, changes in human behaviors and variability of immune function, were also related to the seasonal fluctuations of infectious diseases.

In the present study, we pooled the overall cumulative effect of 6 regions which showed an approximately inverted “V”-shape which was consistent with the results in other studies [31, 32], and reached the maximum cumulative relative risk at 23.8 °C. Our research revealed that warm temperature which ranged from 18 to 28 °C was a hazard factor associated with HFMD. Two studies in Shandong [13] and Shanxi [33] also found that the risk with moderate temperature was the highest. Current studies generally believed that temperature could affect the spread of HFMD. It has been demonstrated that temperature was positively correlated with host activity [34] and the excretion of enteroviruses [35]. The warm conditions helped enteroviruses to survive and multiply in food and drinking water, and appropriate temperature could prolong the survival time of viruses. Therefore, higher temperature increased the exposure of susceptible individuals to source of infection and contaminated environments. At the same time, the increase of temperature could change individual behaviors [8], including eating habits and lifestyle. Higher temperature made people more likely to eat cold dishes, uncooked seafood and ice cream, which could increase the probability of infecting with pathogenic bacteria [36]. Warmer temperature also prompted people to drink large amounts of water, which led to diluted gastric juices and reduced bactericidal defenses, all of which could increase the risk of contracting HFMD. It is well known that temperature and ultraviolet radiation were the two main factors leading to the inactivation of enteroviruses [37]. Therefore, the survival time of enteroviruses was greatly reduced in extreme high temperature environments, which in turn reduced the chance of the host being infected. This could explain the reason why the risk decreased or even showed a negative effect at a very high temperature instead. For the lag effects, the associations of warm temperature with HFMD appeared immediately and lasted longer, indicating that the impact of warm temperature on HFMD needs to be paid attention to. The finding may be related to the fact that temperatures could have influence on the development and longevity of the virus [12, 38]. For instance, the virus could reproduce more rapidly and survive longer at warmer temperature.

From 2010 to 2019 in Chongqing, the number of HFMD cases was 1.39 times higher in males than in females, but the effect of cold temperature on HFMD was slightly higher in females than in males, which was similar to the results of a study in Beijing [9]. There was evidence that women were more vulnerable to cold temperatures than men [39]. The age-specific results showed that children aged 0–1 were more vulnerable to warm temperature and the CRR was the highest, which was consistent with other studies [9, 15]. It has been reported that maternal antibody levels to EV71 declined one month after birth, and thus the lack of immunity increased the susceptibility of children under 1 year of age to temperature [40]. Additionally, the effect of temperature in children aged 3–6 and childcare children showed an approximately M-shape, and temperature ranged from 6 °C to 18 °C was a hazard factor associated with HFMD, with the highest CRR at 10 °C. This may be due to the fact that children aged 3–6 were more likely to gather in a closed environment in cold weather (such as kindergartens), and worse indoor ventilation in cold days might facilitate the transmission of HFMD [41]. However, cold temperature was a protective factor associated with HFMD for scattered children, possibly because they were more likely to stay at home and reduce outdoor activities in cold weather, thus they had less opportunities to contact with each other and be exposed to contaminants [15].

Socio-economic factors, medical and health factors and geo-meteorological factors were taken into account as potential effect modifiers in this study. The results suggested nine modifiers were related to temperature-HFMD relationship. We found that in areas with lower urbanization rates and lower per capita GDP, the warm temperature had a stronger effect on HFMD. According to the study [42], the higher susceptibility of people with lower socioeconomic status may be related to poorer health awareness and vulnerability. Besides, in areas with lower density of health care institutions and lower density of medical technical personnel, the warm temperature had a stronger effect on HFMD. A study [43] had reported that as personal hygiene and sanitation improved in developed countries, the impact of respiratory droplets on virus transmission became less. Poor personal hygiene, inadequate disease knowledge and delayed treatment could increase the risk of disease [44]. In areas where health resources were relatively scarce, it was more difficult to cope with the sudden increase of HFMD cases caused by changes in the external environment such as rising temperatures, which might result in delay in epidemic warning, diagnosis, treatment and other aspects, leading to wider spread of HFMD virus [45]. In addition to the above factors, we found that the meteorological factors could explain a smaller but statistically significant proportion of the spatial heterogeneity. We found that in areas with higher mean temperature, the warm temperature had a weaker effect on HFMD, because continuous high temperature would play a certain role in inactivating the virus. We found that in areas with higher relative humidity, the warm temperature had a stronger effect on HFMD. There was evidence [46] that the survival of virus was directly proportional to the level of relative humidity at the temperature of 20 °C. In addition, it has also been shown that relative humidity was a key influential environmental factor contributing to viral persistence in airborne and foodborne diseases [47]. The discovery in this study that the west region with the highest humidity had a high risk of HFMD in warm temperature also supported this opinion.

The present study also had some limitations. First, the present study selected 38 districts and counties in Chongqing and divided them into 6 regions, so the findings may not be well generalized to other locations in different climatic zones. Second, the modifiers in this study only explained a part of heterogeneity, there were many other modifiers need to be collected. Third, due to data limitations, we did not control the effect of air pollution on HFMD.

## 5. Conclusions

In conclusion, we conducted a two-stage analysis to investigate the effect of temperature on HFMD in 6 regions and further detect the effect modifiers. We found there were nonlinear relationships between temperature and HFMD, with a maximum CRR of 1.22 (95% CI: 1.12–1.34) at 23.8 °C. In general, the warm temperature had a stronger effect on HFMD. Compared with other groups, children aged 0–1 and scattered children were more susceptible to warm temperature, while female, children aged 3–6 and childcare children were more susceptible to cold temperature. Besides, the warm temperature had a stronger effect in areas with lower economic development and poorer health care. The results can also help local authorities take corresponding interventions and measures to control HFMD development before reaching the peak risks.

## Abbreviations

HFMD: Hand, foot, and mouth disease
DLNM: Distributed lag non-linear model
CI: Confidence interval
VIF: Variance inflation factor
CRR: Cumulative relative risk
RR: Relative risk

## Supplementary information

**Additional file 1:** **Table S1** HFMD incidence rate (/100000) in six regions of Chongqing from 2010 to 2019. **Figure S1** Distribution of HFMD cases by year in Chongqing from 2010 to 2019. **Figure S2** Distribution of HFMD cases in Chongqing by temperature from 2010 to 2019.

## Declarations

### Availability of data and material

The data of HFMD cases analyzed in this study were obtained from the Chongqing Center for Disease Control and Prevention, but restrictions apply to the availability of these data, which were used under license for the current study, and so are not publicly available. Data are however available from the authors upon reasonable request and with permission of the above institutions.
