# Supplementary material for: The effect of daily mean temperature on hand, foot and mouth disease and the source of regional heterogeneity in Chongqing, China, 2010–2019
Source: Environ Health Prev Med. 2022 Dec 15;27:47. doi: 10.1265/ehpm.22-00133 (PMC9792571; doi:10.1265/ehpm.22-00133)
Supplement: Supplementary file 1 — Additional file 1: Table S1 HFMD incidence rate (/100000) in six regions of Chongqing from 2010 to 2019. Figure S1 Distribution of HFMD cases by year in Chongqing from 2010 to 2019. Figure S2 Distribution of HFMD cases in Chongqing by temperature from 2010 to 2019. [file ehpm-27-047-s001.docx]

**Table S1 HFMD incidence rate (/100000)** **in six regions of Chongqing from 2010 to 2019**

| Region | | 2010 | 2011 | 2012 | 2013 | 2014 | 2015 | 2016 | 2017 | 2018 | 2019 |
| --- | --- | --- | --- | --- | --- | --- | --- | --- | --- | --- | --- |
| Central urban area | | 176 | 129 | 211 | 187 | 346 | 276 | 372 | 203 | 488 | 343 |
| The west | 32 | 40 | 50 | 78 | 125 | 100 | 149 | 111 | 258 | 152 |  |
| The southwest | 27 | 26 | 25 | 54 | 96 | 101 | 118 | 89 | 200 | 116 |  |
| The middle | 29 | 22 | 49 | 45 | 93 | 121 | 111 | 57 | 194 | 144 |  |
| The southeast | 22 | 14 | 28 | 30 | 98 | 50 | 165 | 80 | 231 | 82 |  |
| The northeast | 38 | 92 | 87 | 90 | 157 | 136 | 183 | 136 | 210 | 194 |  |
| Total | 69 | 70 | 97 | 100 | 184 | 154 | 213 | 132 | 297 | 205 |  |


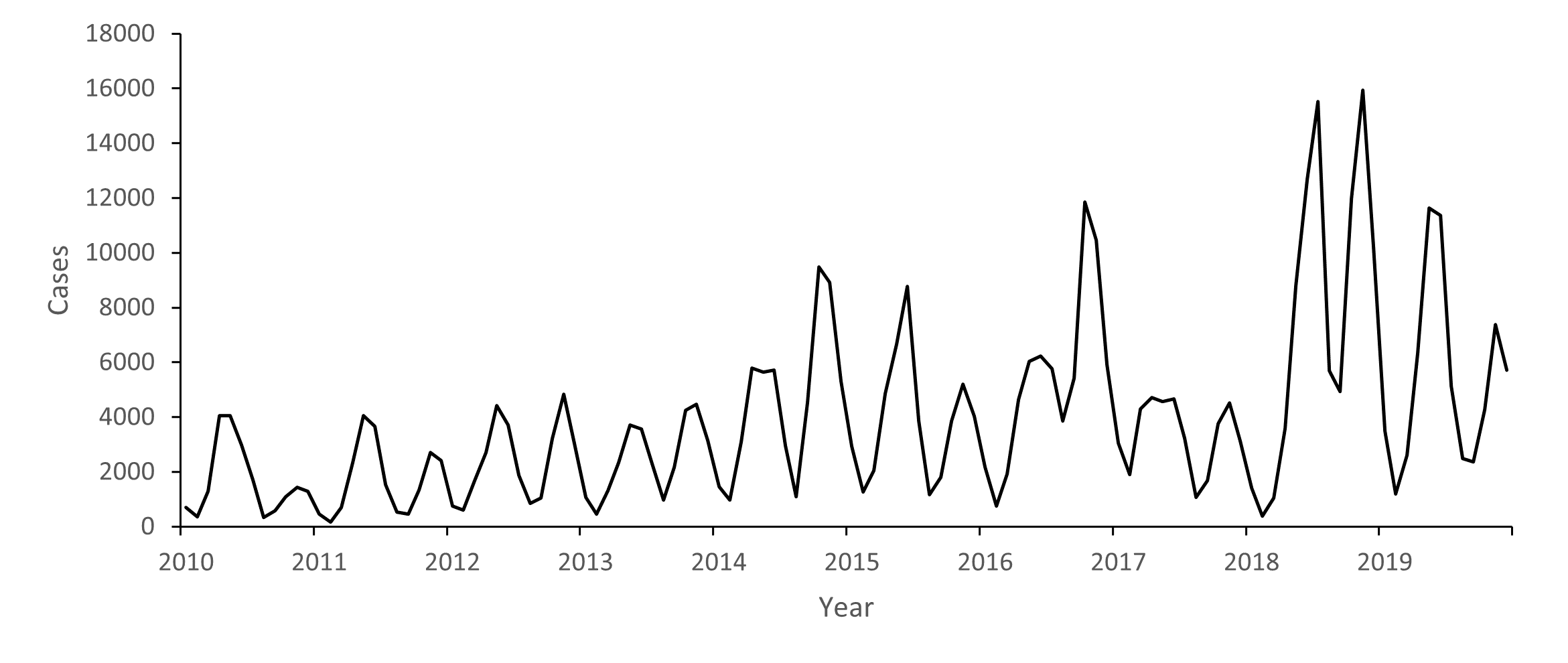


**Figure S1 Distribution of HFMD cases by year in Chongqing from 2010 to 2019**


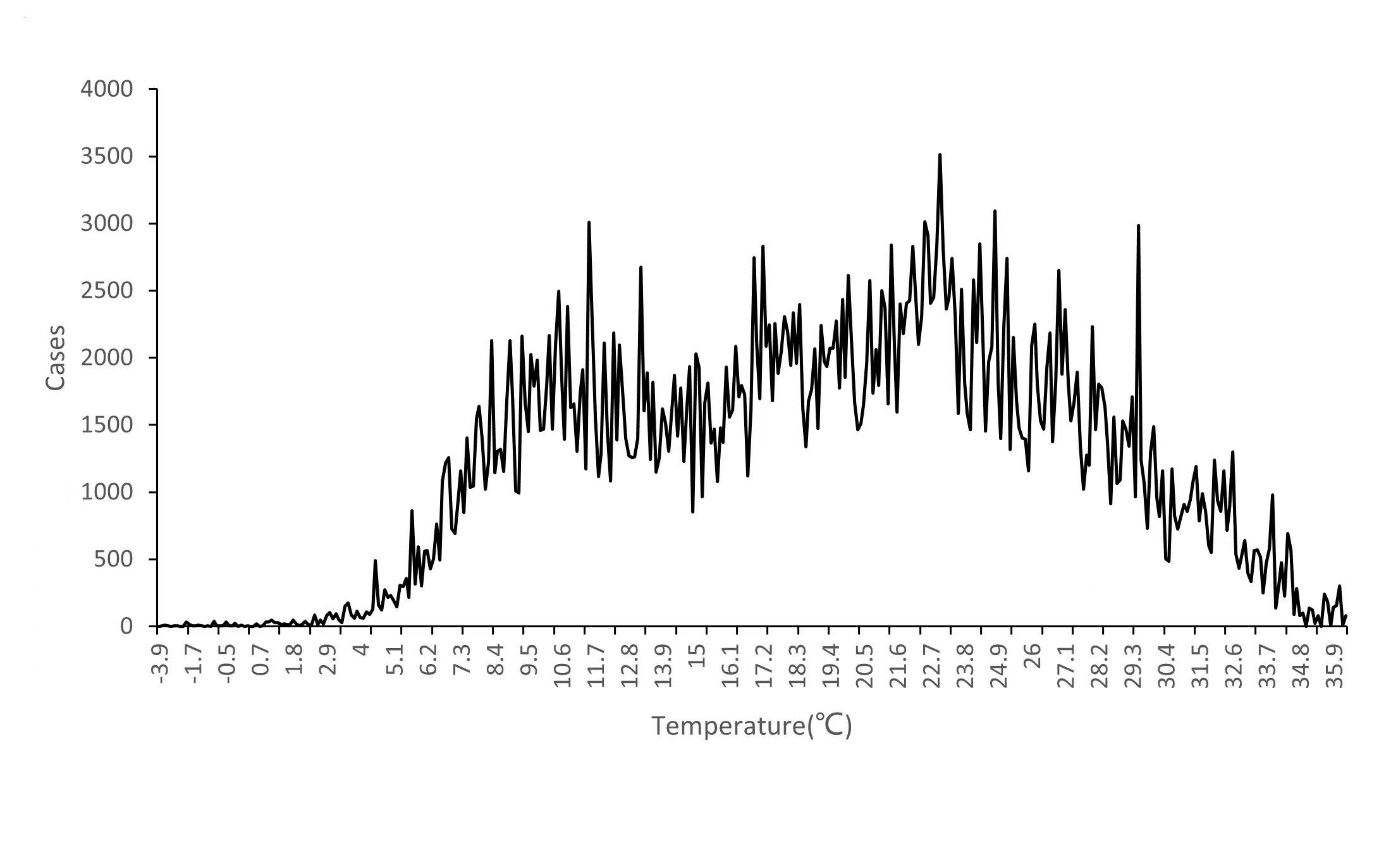


**Figure S2 Distribution of HFMD cases in Chongqing by temperature from 2010 to 2019**
